# Supplementary material for: Sexual coordination in a whole-brain map of prairie vole pair bonding
Source: bioRxiv. 2023 Oct 28:2023.07.26.550685. Originally published 2023 Jul 28. Preprint. [Version 2] doi: 10.1101/2023.07.26.550685 (PMC10402037; doi:10.1101/2023.07.26.550685)
Supplement: Supplement 7 [file NIHPP2023.07.26.550685v2-supplement-7.pdf]

bioRxiv preprint doi: https://doi.org/10.1101/2023.07.26.550685; this version posted October 28, 2023. The copyright holder for this preprint (which was not certified by peer review) is the author/funder, who has granted bioRxiv a license to display the preprint in perpetuity. It is made available under aCC-BY 4.0 International license.

Figure supplements

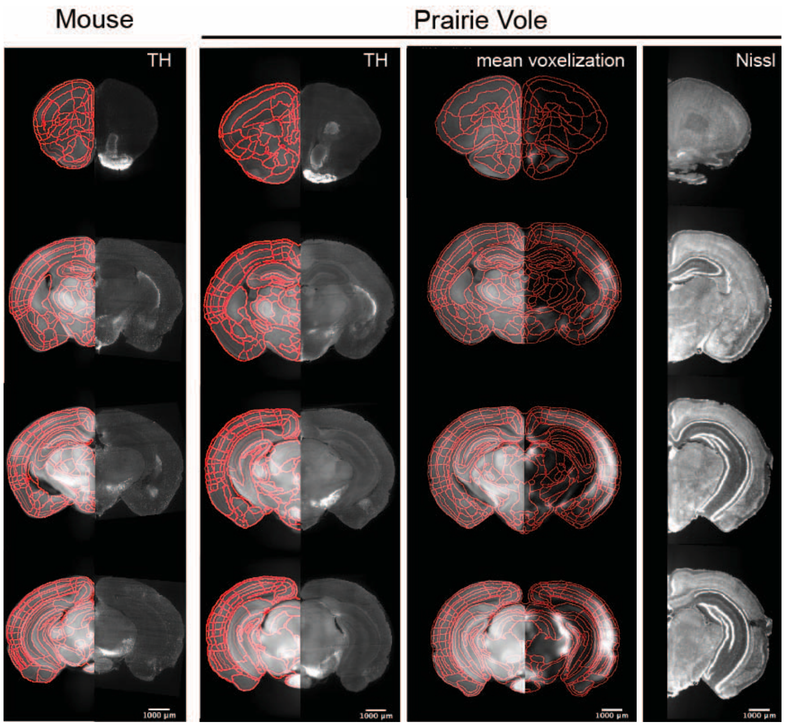

Figure 1—figure supplement 1. Validation of the prairie vole reference atlas. Mouse coronal sections (left column) are composed of the mouse reference brain overlaid with atlas boundaries in red on the left and, in the same sections, of tyrosine hydroxylase (TH) immunolabeling on the right. Prairie vole coronal sections are in the right three columns. In the first vole column, coronal brain sections are of the prairie vole reference brain and atlas boundaries in red on the left with TH immunolabeling on the right. In the second vole column, coronal sections are of the prairie vole reference brain and c-Fos+ mean voxelization overlay with atlas boundaries in red. In the third vole column, coronal sections of prairie vole NeuroTrace staining are registered onto the prairie vole reference brain.

bioRxiv preprint doi: https://doi.org/10.1101/2023.07.26.550685; this version posted October 28, 2023. The copyright holder for this preprint (which was not certified by peer review) is the author/funder, who has granted bioRxiv a license to display the preprint in perpetuity. It is made available under aCC-BY 4.0 International license.

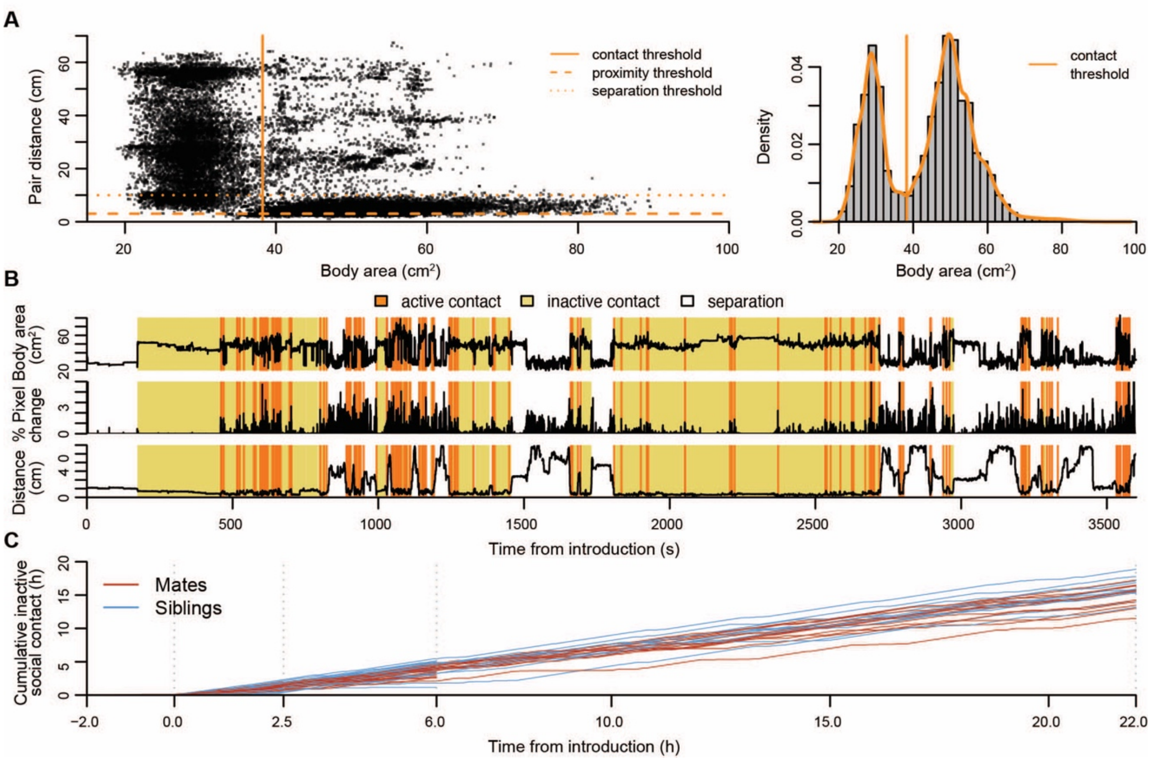

Figure 2—figure supplement 1. Automated tracking of social behavior states. (A) Long-term tracking of social states was informed by automated measures of the largest body area, pair activity, and pair distance. On the left, body area is plotted against dyad distance from an exemplar 24h mate pair video (10% randomly selected frames from 12 hours of white light). On the right, the density curve for body area reveals a basic threshold for when animals are separated or in physical contact. **(B)** Traces of body area, video activity, and pair distance are plot for the first hour of interaction of the exemplar mate pair, along with automated assignments of behavioral states. **(C)** Cumulative time spent in inactive social contact for up to 22h of cohabitation in mate pairs (red lines) and sibling pairs (blue lines).

bioRxiv preprint doi: https://doi.org/10.1101/2023.07.26.550685; this version posted October 28, 2023. The copyright holder for this preprint (which was not certified by peer review) is the author/funder, who has granted bioRxiv a license to display the preprint in perpetuity. It is made available under aCC-BY 4.0 International license.

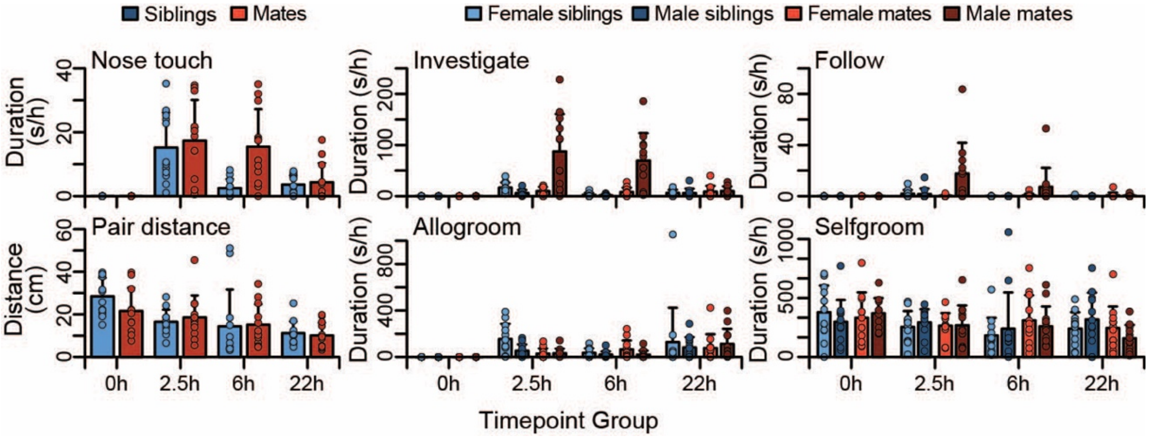

Figure 2—figure supplement 2. Time course of social behaviors during pairing. Group differences (mean ± sd) are shown for appetitive behaviors including nose-to-nose touching, anogenital investigation, and close follows. Group differences (mean ± sd) are shown for proximity and grooming behaviors including pair distance, allogrooming, and selfgrooming. Mate pairs are in red and sibling pairs in blue (female in lighter hues, males in darker hues). T-tests were used to compare mates and siblings, and paired t-tests were used to compare female and male mates.

bioRxiv preprint doi: https://doi.org/10.1101/2023.07.26.550685; this version posted October 28, 2023. The copyright holder for this preprint (which was not certified by peer review) is the author/funder, who has granted bioRxiv a license to display the preprint in perpetuity. It is made available under aCC-BY 4.0 International license.

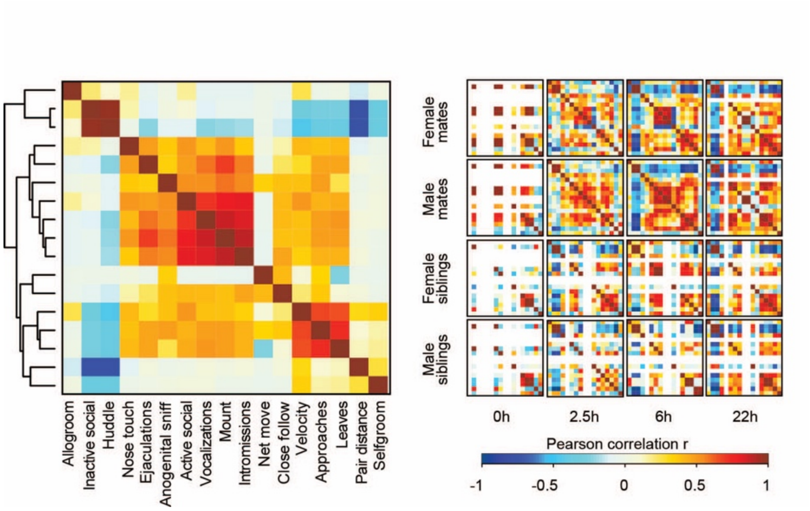

Figure 2—figure supplement 3. Associations between behavioral states and types of social interaction. Hierarchical clustering of behavioral measures from Pearson correlations groups behavioral states and interactions into three main clusters involving close contact (e.g., huddling and allogrooming), mating (e.g., mounts, vocalizations), and appetitive behavior (e.g., approaches and follows). On the right, Pearson correlations are shown among behaviors in subsets based on partner type, sex, and timepoint. Warm and cool colors indicate positive and negative correlation coefficients, respectively. White indicates behaviors with no variation, meaning that coefficients were not computed (e.g., siblings did not exhibit mating behaviors).

bioRxiv preprint doi: https://doi.org/10.1101/2023.07.26.550685; this version posted October 28, 2023. The copyright holder for this preprint (which was not certified by peer review) is the author/funder, who has granted bioRxiv a license to display the preprint in perpetuity. It is made available under aCC-BY 4.0 International license.

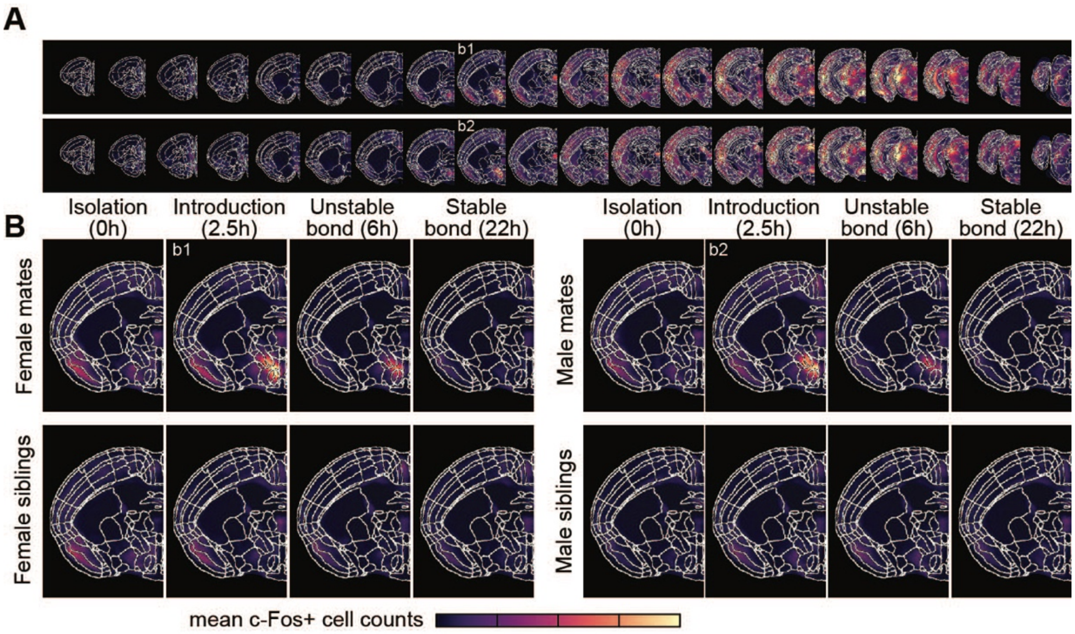

Figure 3—figure supplement 1. Brain-wide patterns of immediate early gene activation during pairing. **(A)** Coronal cross-sections (rostral to caudal) from female (top) and male (bottom) mating pairs are shown for the 2.5h timepoint group, with brightness corresponding to the average voxel c-Fos+ cell counts. **(B)** A representative coronal slice that includes posterior BST is shown for mate pairs and siblings and separated by timepoint and sex. Brightness corresponds to average voxel c-Fos+ cell counts per group.

bioRxiv preprint doi: https://doi.org/10.1101/2023.07.26.550685; this version posted October 28, 2023. The copyright holder for this preprint (which was not certified by peer review) is the author/funder, who has granted bioRxiv a license to display the preprint in perpetuity. It is made available under aCC-BY 4.0 International license.

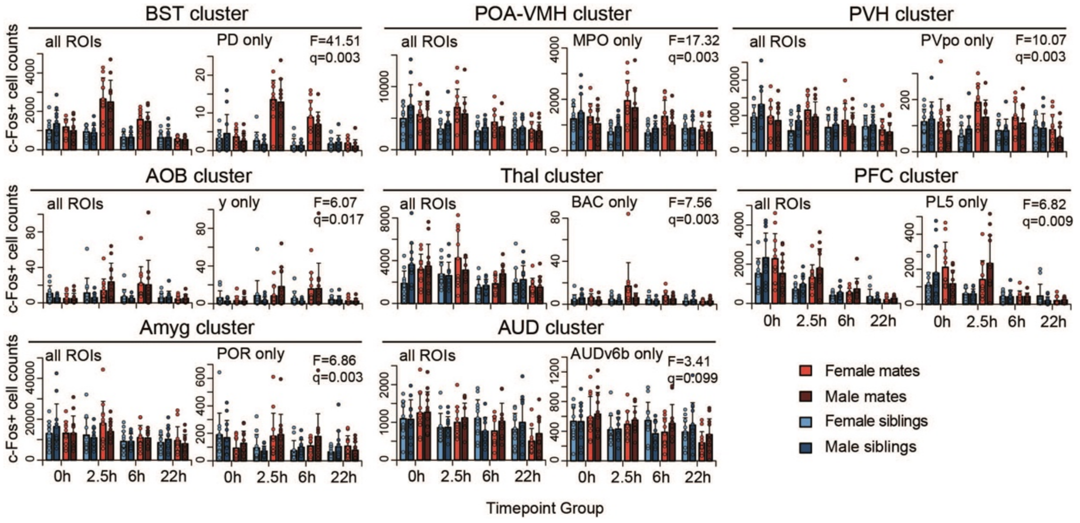

Figure 3—figure supplement 2. Patterns of immediate early gene expression in brain region clusters. Counts of c-Fos+ cells are shown for ROIs associated with pair bond development, organized by hierarchal cluster. For each cluster, the total counts are shown on the right and the most significant ROI (highest F-statistic) is shown on the right. Counts are summarized by timepoint, partner type and sex (mean ± sd), and overlaid with counts from individual animals. Mate pairs are in red and sibling pairs in blue (female in lighter hues, males in darker hues). Test statistics are from an ANOVA to compare null and hypothesized general linear models. FDR q-values are from permutation tests to assess the likelihood of the observed test statistic as compared to a distribution of test statistics obtained from shuffled data of 10,000 permutations.

bioRxiv preprint doi: https://doi.org/10.1101/2023.07.26.550685; this version posted October 28, 2023. The copyright holder for this preprint (which was not certified by peer review) is the author/funder, who has granted bioRxiv a license to display the preprint in perpetuity. It is made available under aCC-BY 4.0 International license.

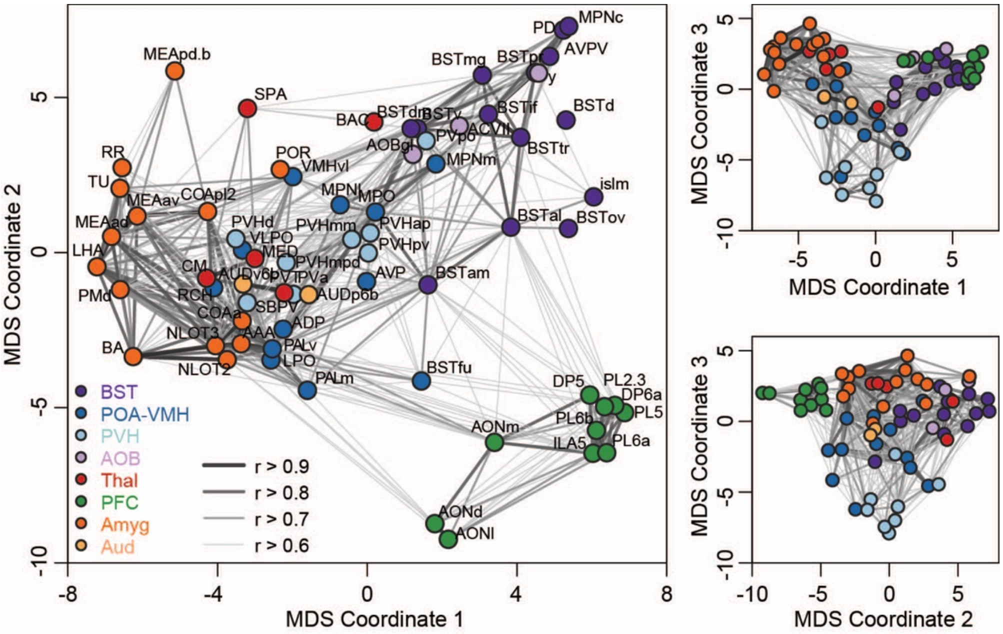

Figure 3—figure supplement 3. Multi-dimensional structure of brain-wide correlation patterns. Representations of the first three dimensions of a multi-dimensional scaling (MDS) coordinate space based on Pearson correlations between c-Fos+ cell counts in brain regions (ROIs) associated with bonding. Each symbol represents an ROI and is colored based on cluster assignment. Darkness and thickness of connecting lines reflect correlation coefficients.

bioRxiv preprint doi: https://doi.org/10.1101/2023.07.26.550685; this version posted October 28, 2023. The copyright holder for this preprint (which was not certified by peer review) is the author/funder, who has granted bioRxiv a license to display the preprint in perpetuity. It is made available under aCC-BY 4.0 International license.

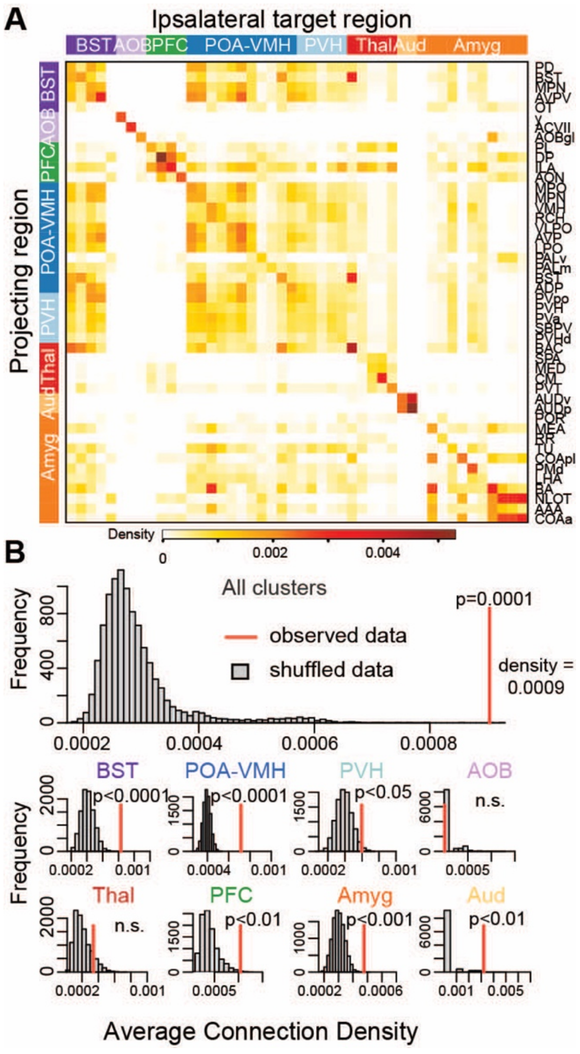

Figure 3—figure supplement 4. Anatomical connectivity in brain regions associated with pairing. (A) This heatmap shows normalized connection densities (Knox et al., 2019) from projecting to ipsilateral target ROIs in the mouse brain. These ROIs are the same as, or larger divisions that contain, the ROIs found to be associated with pair bond development in our analysis. **(B)** The top histogram shows results of a permutation test to assess whether hierarchical clusters of chosen ROIs in our analysis mirror underlying anatomical connections. Observed connectivity reflects the overall average of cluster means, which is compared to averages from 10,000 iterations of shuffled data (i.e., target regions shuffled for each projecting region). The bottom histograms show the results of permutation tests for the mean connectivity within each cluster of ROIs.

bioRxiv preprint doi: https://doi.org/10.1101/2023.07.26.550685; this version posted October 28, 2023. The copyright holder for this preprint (which was not certified by peer review) is the author/funder, who has granted bioRxiv a license to display the preprint in perpetuity. It is made available under aCC-BY 4.0 International license.

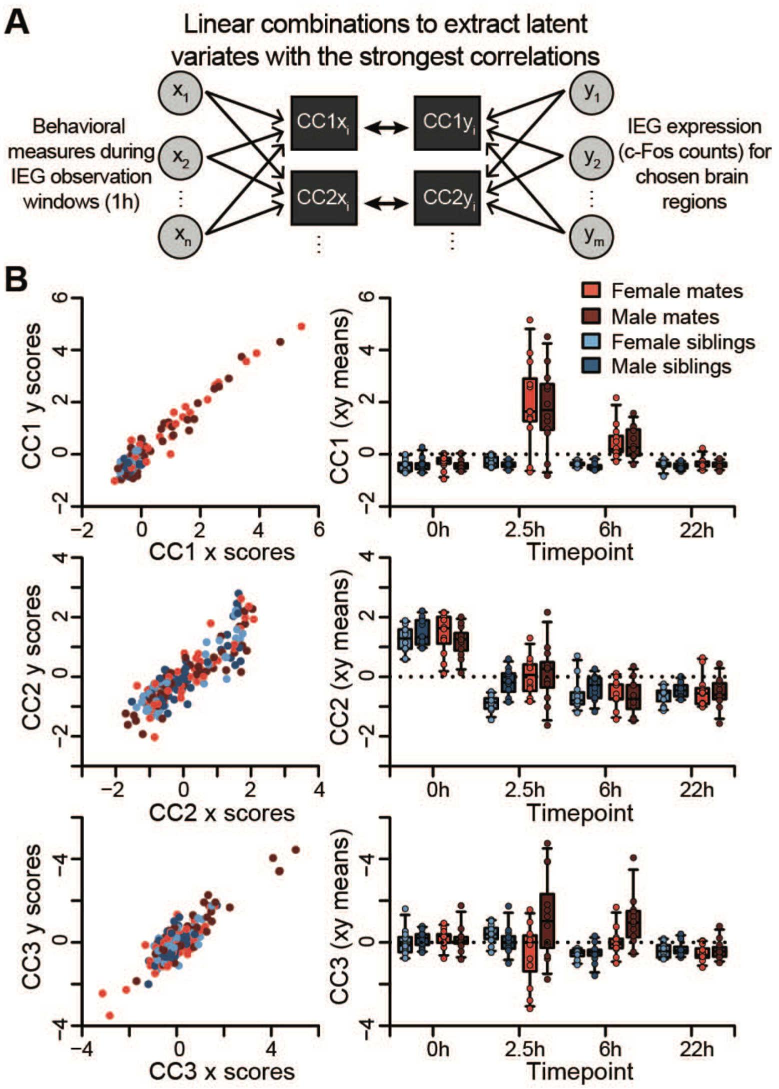

Figure 4—figure supplement 1. Dimensions of cross-covariance in immediate early gene expression and social behavior. (A) Schematic of canonical correlation analysis (CCA), where two sets of variables (x and y) are combined linearly to extract latent variates with the strongest correlations (CC1 stronger than CC2, and so on). This analysis outputs x and y scores for each latent variate for each animal subject in the dataset. In this dataset, x scores represent linear combinations of behavioral measures, and y scores represent linear combinations of ROI c-Fos counts. **(B)** On the left, the relationship between x and y scores is shown for all study animals for the first three variates. On the right, the means of x and y scores for the first three variates are compared across partner type, sex, and timepoint (boxplots = median, 25%-75% quartile, and 95% confidence intervals).

bioRxiv preprint doi: https://doi.org/10.1101/2023.07.26.550685; this version posted October 28, 2023. The copyright holder for this preprint (which was not certified by peer review) is the author/funder, who has granted bioRxiv a license to display the preprint in perpetuity. It is made available under aCC-BY 4.0 International license.

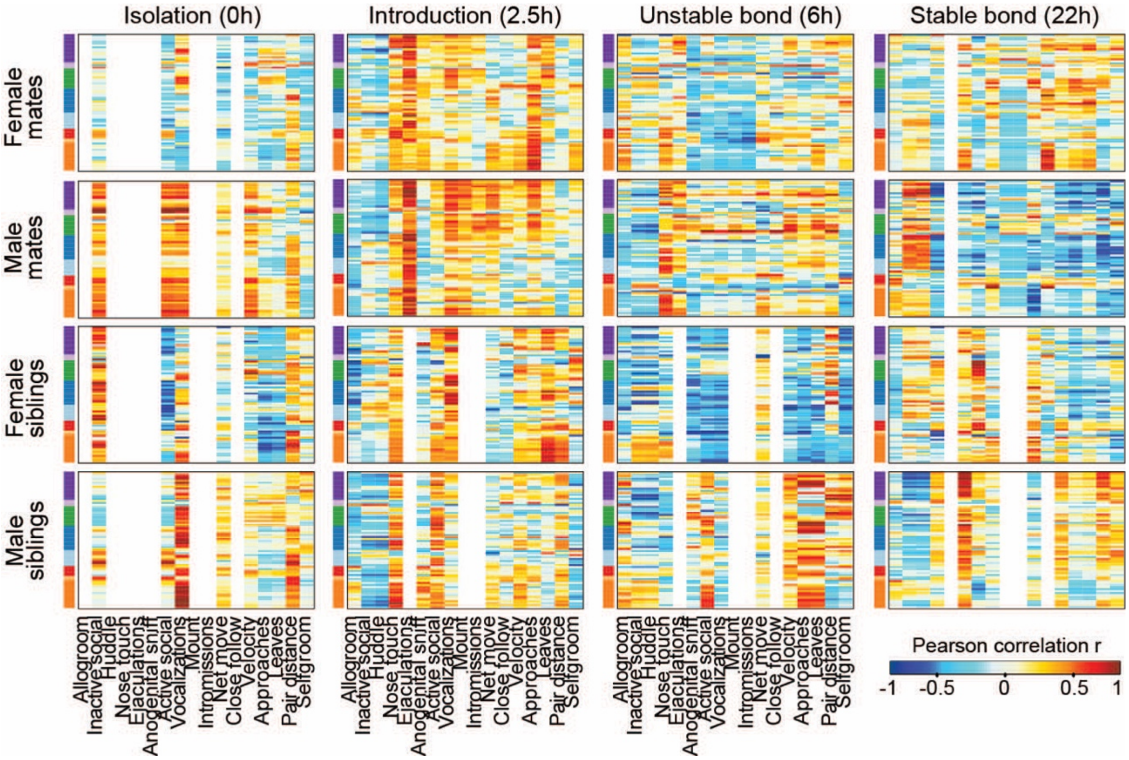

Figure 4—figure supplement 2. Intra-pair similarity in immediate early gene expression in pairing-associated brain regions. Heatmaps represent Pearson correlation coefficients, with brain regions on the y-axis (grouped into hierarchical clusters, see Figure 3 for ROI and cluster labels) and behavioral outcomes (during 1h observation windows) on the x-axis. Correlation heatmaps are split by partner type, sex and timepoint. Warm and cool colors indicate positive and negative correlation coefficients, respectively. White indicates behaviors with no variation, meaning that coefficients were not computed (e.g., siblings did not exhibit mating behaviors).

bioRxiv preprint doi: https://doi.org/10.1101/2023.07.26.550685; this version posted October 28, 2023. The copyright holder for this preprint (which was not certified by peer review) is the author/funder, who has granted bioRxiv a license to display the preprint in perpetuity. It is made available under aCC-BY 4.0 International license.

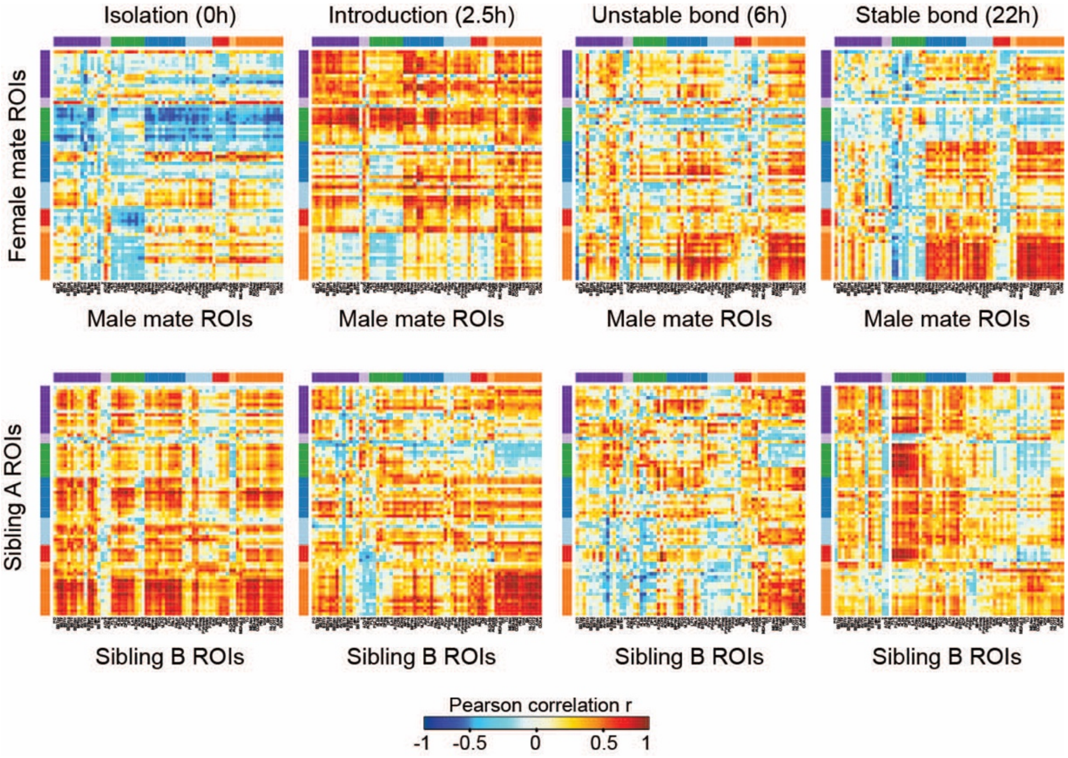

Figure 4—figure supplement 3. Patterns of association between immediate early gene expression and behavior. On the top, heatmaps represent Pearson correlation coefficients between selected brain regions (ROIs) in mating pairs, with female data on the y-axis and male data on the x-axis. On the bottom, heatmaps represent Pearson correlation coefficients between selected ROIs in sibling dyads, where sibling A data are from animals placed in the left side of chamber during acclimation, and sibling B data are from animals placed on the right side. ROIs are grouped by hierarchical clustering (see Figure 3). Warm and cool colors indicate positive and negative correlation coefficients, respectively.
