# Supplementary material for: Sexual coordination in a whole-brain map of prairie-vole pair-bonding
Source: bioRxiv. 2023 Jul 28:2023.07.26.550685. Preprint. [Version 1] doi: 10.1101/2023.07.26.550685 (PMC10402037; doi:10.1101/2023.07.26.550685)
Supplement: Supplementary file 1 [file NIHPP2023.07.26.550685v1-supplement-8.pdf]

bioRxiv preprint doi: https://doi.org/10.1101/2023.07.26.550685; this version posted July 28, 2023. The copyright holder for this preprint (which was not certified by peer review) is the author/funder, who has granted bioRxiv a license to display the preprint in perpetuity. It is made available under aCC-BY 4.0 International license.

Supplementary information

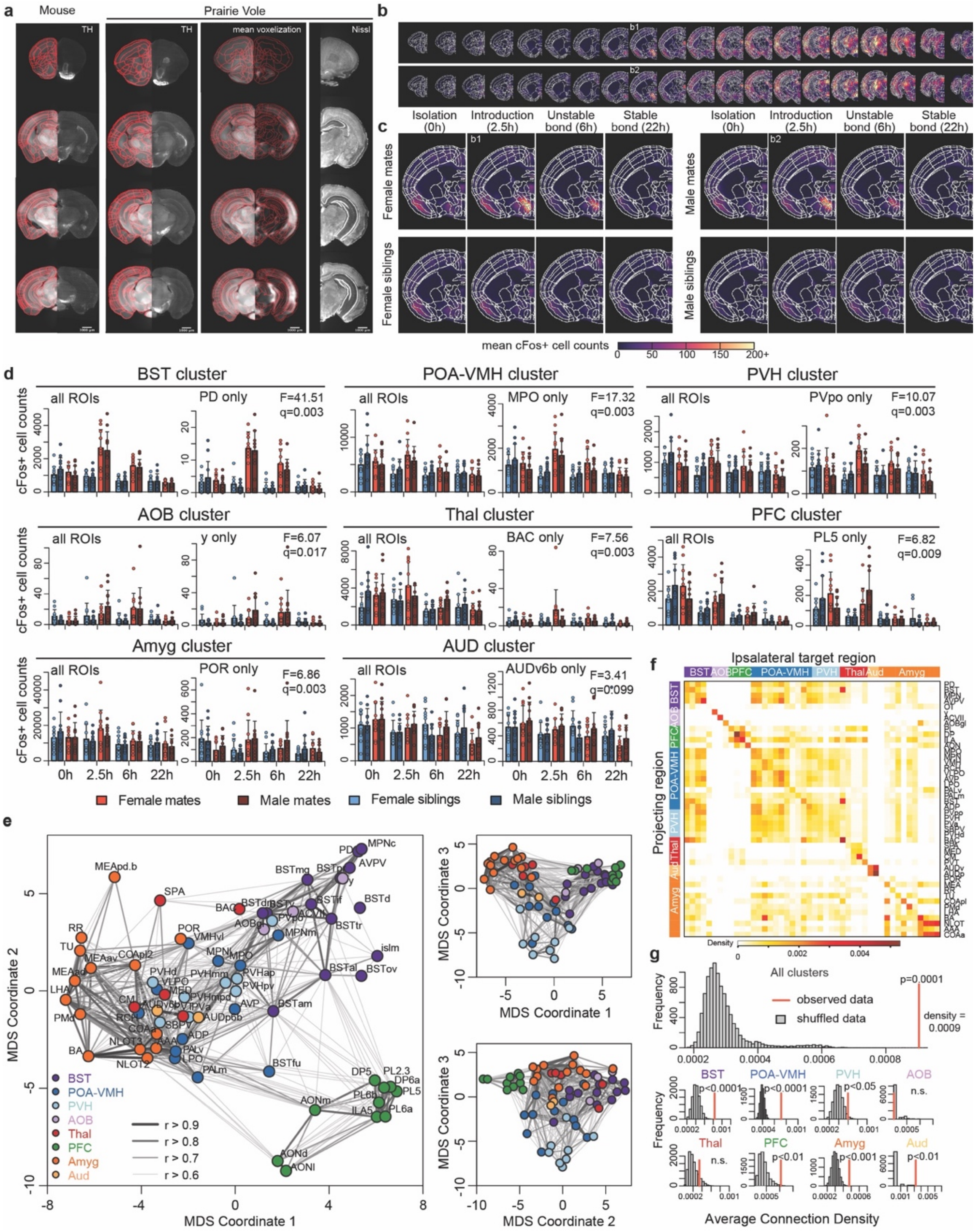

Figure Supplement 1. Validation of the prairie vole reference atlas and brain-wide patterns of immediate early gene expression during pairing. a. Mouse (left panel). Coronal sections composition of mouse reference brain overlay with atlas boundaries in red (left) and the same

bioRxiv preprint doi: https://doi.org/10.1101/2023.07.26.550685; this version posted July 28, 2023. The copyright holder for this preprint (which was not certified by peer review) is the author/funder, who has granted bioRxiv a license to display the preprint in perpetuity. It is made available under aCC-BY 4.0 International license.

section of TH immunolabeling (right). Prairie vole (left column). Similar coronal brain sections of prairie vole reference brain and atlas boundaries in red (left) with TH immunolabeling (right). (center column) Same coronal section of prairie vole reference brain and c-Fos+ mean voxelization overlay with atlas boundaries in red. (left column) Same coronal sections of prairie vole NeuroTrace staining registered onto prairie vole reference brain. **b.** Coronal cross-sections (rostral to caudal) from female (top) and male (bottom) mating pairs are shown for the 2.5h timepoint group, with brightness corresponding to the average voxel c-Fos+ cell counts. **c.** A representative coronal slice that includes posterior BST is shown for mate pairs and siblings and separated by timepoint and sex. Brightness corresponds to average voxel c-Fos+ cell counts per group. **d.** Counts of c-Fos+ cells are shown for ROIs associated with pair-bond development, organized by hierarchal cluster. For each cluster, the total counts are shown on the right and the most significant ROI (highest F-statistic) is shown on the right. Counts are summarized by timepoint, partner type and sex (mean ± sd), and overlaid with counts from individual animals. Mate pairs are in red and sibling pairs in blue (female in lighter hues, males in darker hues). Test statistics are from an ANOVA to compare null and hypothesized general linear models. FDR q-values are from permutation tests to assess the likelihood of the observed test statistic as compared to a distribution of test statistics obtained from shuffled data. **e.** Representations of the first three dimensions of a multi-dimensional scaling (MDS) coordinate space based on Pearson correlations between c-Fos+ cell counts in brain regions (ROIs) associated with bonding. Each symbol represents an ROI and is colored based on cluster assignment. Darkness and thickness of connecting lines reflect correlation coefficients. **f.** This heatmap shows normalized connection densities (Knox et al., 2019) from projecting to ipsilateral target ROIs in the mouse brain. These ROIs are the same as, or larger divisions that contain, the ROIs found to be associated with pair-bond development in our analysis. **g.** The top histogram shows results of a permutation test to assess whether hierarchical clusters of chosen ROIs in our analysis mirror underlying anatomical connections. Observed connectivity reflects the overall average of cluster means, which is compared to averages from 10,000 iterations of shuffled data (i.e., target regions shuffled for each projecting region). The bottom histograms show the results of permutation tests for the mean connectivity within each cluster of ROIs.

bioRxiv preprint doi: https://doi.org/10.1101/2023.07.26.550685; this version posted July 28, 2023. The copyright holder for this preprint (which was not certified by peer review) is the author/funder, who has granted bioRxiv a license to display the preprint in perpetuity. It is made available under aCC-BY 4.0 International license.

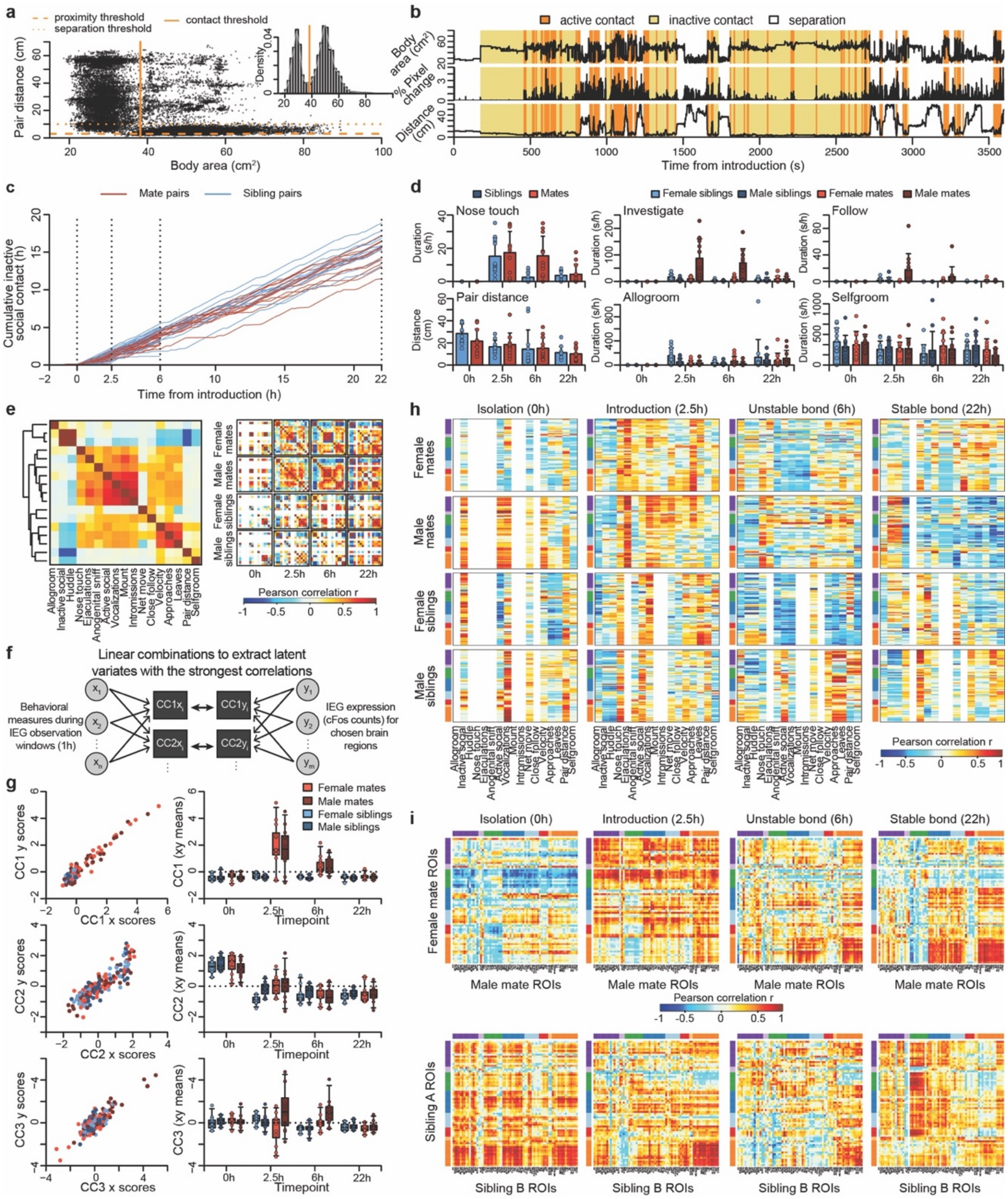

Figure Supplement 2. Behavioral phenotypes during pairing and patterns of associate between brain-wide immediate early gene expression and behavior. a. Long-term tracking of social states was informed by automated measures of the largest body area, pair activity, and pair distance. On the left, body area is plotted against dyad distance from an exemplar 24h mate pair video (10% randomly selected frames from 12 hours of white light). On the right, the density curve for body area reveals a basic threshold for when animals are separated or in physical

bioRxiv preprint doi: https://doi.org/10.1101/2023.07.26.550685; this version posted July 28, 2023. The copyright holder for this preprint (which was not certified by peer review) is the author/funder, who has granted bioRxiv a license to display the preprint in perpetuity. It is made available under aCC-BY 4.0 International license.

contact. **b.** Traces of body area, video activity, and pair distance are plot for the first hour of interaction of the exemplar mate pair, along with automated assignments of behavioral states. **c.** Cumulative time spent in inactive social contact for up to 22h of cohabitation in mate pairs (red lines) and sibling pairs (blue lines). **d.** Group differences (mean ± sd) are shown for appetitive behaviors including nose-to-nose touching, anogenital investigation, and close follows. Group differences (mean ± sd) are shown for proximity and grooming behaviors including pair distance, allogrooming, and selfgrooming. Mate pairs are in red and sibling pairs in blue (female in lighter hues, males in darker hues). T-tests were used to compare mates and siblings, and paired t-tests were used to compare female and male mates. **e.** Hierarchical clustering of behavioral measures from Pearson correlations groups behavioral states and interactions into three main clusters involving close contact (e.g., huddling and allogrooming), mating (e.g., mounts, vocalizations), and appetitive behavior (e.g., approaches and follows). On the right, Pearson correlations are shown among behaviors in subsets based on partner type, sex, and timepoint. Warm and cool colors indicate positive and negative correlation coefficients, respectively. White indicates behaviors with no variation, meaning that coefficients were not computed (e.g., siblings did not exhibit mating behaviors). **f.** Schematic of canonical correlation analysis (CCA), where two sets of variables (x and y) are combined linearly to extract latent variates with the strongest correlations (CC1 stronger than CC2, and so on). This analysis outputs x and y scores for each latent variate for each animal subject in the dataset. In this dataset, x scores represent linear combinations of behavioral measures, and y scores represent linear combinations of ROI c-Fos counts. **g.** On the left, the relationship between x and y scores is shown for all study animals for the first three variates. On the right, the means of x and y scores for the first three variates are compared across partner type, sex, and timepoint (boxplots = median, 25%-75% quartile, and 95% confidence intervals). **h.** Heatmaps represent Pearson correlation coefficients, with brain regions on the y-axis (grouped into hierarchical clusters, see Figure 3 for ROI and cluster labels) and behavioral outcomes (during 1h observation windows) on the x-axis. Correlation heatmaps are split by partner type, sex and timepoint. Warm and cool colors indicate positive and negative correlation coefficients, respectively. White indicates behaviors with no variation, meaning that coefficients were not computed (e.g., siblings did not exhibit mating behaviors). **i.** On the top, heatmaps represent Pearson correlation coefficients between selected brain regions (ROIs) in mating pairs, with female data on the y-axis and male data on the x-axis. On the bottom,

bioRxiv preprint doi: https://doi.org/10.1101/2023.07.26.550685; this version posted July 28, 2023. The copyright holder for this preprint (which was not certified by peer review) is the author/funder, who has granted bioRxiv a license to display the preprint in perpetuity. It is made available under aCC-BY 4.0 International license.

heatmaps represent Pearson correlation coefficients between selected ROIs in sibling dyads, where sibling A data are from animals placed in the left side of chamber during isolation, and sibling B data are from animals placed on the right side. ROIs are grouped by hierarchical clustering (see Figure 3). Warm and cool colors indicate positive and negative correlation coefficients, respectively.

Supplemental File 1. Statistical comparisons of absolute and relative brain area volumes between prairie voles and mice. Absolute and relative area volumes (normalized to total brain volume) are compared between prairie voles and male mice. Absolute brain area volumes are all statistically significant bigger in the prairie vole compared to the mouse, but none the ratios of area volume (relative to the whole brain) were statistically different between species. Mean, SD, p-values, and FDR correction of the p-values are presented for each region.

Supplemental File 2 (separate file). Statistical comparisons of absolute and relative brain area volumes between male and female prairie voles. Absolute and relative area volumes (normalized to total brain volume) are compared between male and female prairie voles. No statistical differences were found in these sex comparisons. Mean, SD, p-values, and FDR correction of the p-values are presented for each region.

Supplemental File 3 (separate file). Behavior ethogram. Descriptions of automated, semi-automated, and manually scored behavioral measures.

Video 1. Prairie vole reference brain. The prairie vole reference brain from LSFM imaging is shown from coronal cross-sections (rostral to caudal) and in a 3D view. Then, coronal cross-sections are show of the prairie vole atlas (left) alongside whole-brain staining for somatostatin (SST, middle) and fluorescent Nissl (NeuroTrace, right).

bioRxiv preprint doi: https://doi.org/10.1101/2023.07.26.550685; this version posted July 28, 2023. The copyright holder for this preprint (which was not certified by peer review) is the author/funder, who has granted bioRxiv a license to display the preprint in perpetuity. It is made available under aCC-BY 4.0 International license.

Video 2. Patterns of whole-brain immediate early gene expression during pairing. First, coronal cross-sections of average IEG induction patterns are shown for female (first video) and male (second video) prairie voles across each of the 16 experiment groups split by partner type (mates-top, siblings-bottom) and timepoint (0, 2.5, 6, 22 hours from left to right). Brightness corresponds to average voxel c-Fos+ cell counts. These videos are associated with Figure Supplement 1b,c (see these panels for the color key). The induction patterns are overlaid with prairie vole atlas boundaries. Next, coronal cross-sections are shown with the results of model comparisons to identify brain areas associated with bonding. This video is associated with Figure 3A (see this panel for the color key). The reference is overlaid with colored voxels that represent a difference (FDR $q < 0.1$) between hypothesized general linear model that included partner type and a null model that did not. Warm colors indicate voxels with higher c-Fos+ counts in mate pairs (test statistic for partner type > 0), and cooler colors indicate voxels with higher c-Fos+ cell counts in siblings (test statistic for partner type < 0). Finally, coronal cross-sections are shown with the results of model comparisons to identify brain areas associated with sex differences. The reference is overlaid with colored voxels that represent a difference (FDR $q < 0.1$) between hypothesized general linear model that included a partner type x sex interaction term and a null model that did not. Colored voxels indicate regions where there was an interaction effect, with warm colors indicating a positive interaction term and cool colors indicating a negative interaction term (see Figure 3A color key).
